# Cigarette Smoke Upregulates Rat Coronary Artery Endothelin Receptors In Vivo

**DOI:** 10.1371/journal.pone.0033008

**Published:** 2012-03-07

**Authors:** Lei Cao, Yaping Zhang, Yong-Xiao Cao, Lars Edvinsson, Cang-Bao Xu

**Affiliations:** 1 Division of Experimental Vascular Research, Institute of Clinical Science in Lund, Lund University, Lund, Sweden; 2 Department of Pharmacology, Xi'an Jiaotong University College of Medicine, Xi'an, Shaanxi, People's Republic of China; University of Giessen Lung Center, Germany

## Abstract

**Background:**

Cigarette smoking is a strong cardiovascular risk factor and endothelin (ET) receptors are related to coronary artery diseases. The present study established an *in vivo* secondhand smoke (SHS) exposure model and investigated the hypothesis that cigarette smoke induces ET receptor upregulation in rat coronary arteries and its possible underlying mechanisms.

**Methodology/Principal Findings:**

Rats were exposed to SHS for 200 min daily for 8 weeks. The coronary arteries were isolated and examined. The vasoconstriction was studied by a sensitive myograph. The expression of mRNA and protein for receptors was examined by real-time PCR, Western blot and immunofluorescence. Compared to fresh air exposure, SHS increased contractile responses mediated by endothelin type A (ET_A_) and type B (ET_B_) receptors in coronary arteries. In parallel, the expression of mRNA and protein for ET_A_ and ET_B_ receptors of smoke exposed rats were higher than that of animals exposed to fresh air, suggesting that SHS upregulates ET_A_ and ET_B_ receptors in coronary arteries *in vivo*. Immunofluorescence staining showed that the enhanced receptor expression was localized to the smooth muscle cells of coronary arteries. The protein levels of phosphorylated (p)-Raf-1 and p-ERK1/2 in smoke exposed rats were significantly higher than in control rats, demonstrating that SHS induces the activation of the Raf/ERK/MAPK pathway. Treatment with Raf-1 inhibitor GW5074 suppressed SHS-induced enhanced contraction mediated by ET_A_ receptors, and inhibited the elevated mRNA and protein levels of ET_A_ and ET_B_ receptors caused by SHS. The results of correlation and regression analysis showed that phosphorylation of Raf and ERK1/2 were independent determinants to affect protein expression of ET_B_ and ET_A_ receptors.

**Conclusions/Significance:**

Cigarette smoke upregulates ET_B_ and ET_A_ receptors in rat coronary artery, which is associated with the activation of the Raf/ERK/MAPK pathway.

## Introduction

Cigarette smoking is considered one of the most powerful risk factors for the development of cardiovascular disease (CVD), such as coronary artery disease (CAD), ischemic and congestive heart failure [1]. Increasing evidences demonstrate that passive smoke (secondhand smoke, SHS) contains relatively higher concentrations of toxic gaseous components than mainstream smoke [2]. Smoking is strongly associated with the pathogenesis of CAD which may be attributed to the promotion of atherosclerosis, the triggering of coronary thrombosis, coronary artery spasm and reduced capacity of the blood to deliver oxygen [3]. Although cigarette smoking is an established risk factor for atherosclerosis, the actual mediator of CAD associated with smoking is unknown.

The endothelin system plays an important role in CVD pathogenesis. Endothelin-1 (ET-1) is one of the important vasoactive peptide participating in the regulation of vascular functions, such as controlling the basal coronary artery tone [4]. To exert its effects, ET-1 binds to transmembrane endothelin type A (ET_A_) and type B (ET_B_) receptors to mediate vasomotor responses [5] which can alter the diameter of the vessel lumen as well as blood flow in the vasculature [6]. The ET_A_ and ET_B_ receptors in the vascular smooth muscle cells (VSMC) are involved in the atherosclerotic process by enhancing proliferation and migration of the VSMC [7]. In addition, there are many observations of ET_B_ receptor upregulation in patients [8] and experimental models [9] of atherosclerosis and ischemic heart disease [10].

The ET receptor system has been shown to be involved in the pathophysiological events that are associated with cigarette smoking [11]. By using an *in vitro* model, we have shown that dimethylsulfoxide-soluble smoke particles induce upregulation of ET receptors in rat cerebral [12] and mesenteric [13] arteries. Recently, a novel aspect of the vascular pathophysiology has been revealed, namely the upregulation of vasoconstrictor receptors in the smooth muscle of arteries [14]. There are differences in receptor expression depending on different arteries, which may occur as a result in various diseases. As we know, receptor changes in cerebral arteries are related to ischemic stroke [14]. Receptor expression alterations in coronary arteries are associated with CAD and/or atherosclerosis [8], [10]. Likewise, the coronary artery also possesses ET receptors [15]. Therefore, we hypothesize that cigarette smoking or SHS upregulates coronary artery ET receptors, which may be of considerable relevance to the understanding of SHS-associated CAD and/or atherosclerosis.

The mitogen-activated protein kinases (MAPKs) are serine/threonine-specific protein kinases that respond to extracellular stimuli and regulate various cellular activities [16]. MAPKs consist of three main signaling pathways: extracellular signal-regulated protein kinase 1 and 2 (ERK1/2), c-Jun N-terminal kinase (JNK) and p38 [17]. Raf-1 is the initial protein kinase in MAPK signal transduction and takes turns to phosphorylate the subsequent MAP kinase/ERK (MEK) 1/2 and ERK1/2 [18]. The MAPK signaling pathways have been shown to be associated with the process of receptor upregulation in human and rat vasculatures [19], [20]. Recent studies have demonstrated that the ET_B_ and ET_A_ receptor upregulation can be attenuated by using a MEK/ERK inhibitor [21], supporting that there is a tight correlation between MEK/ERK pathway and ET receptor upregulation.

The present study established an *in vivo* SHS exposure model and investigated the hypothesis that cigarette smoke induces ET receptor upregulation in rat coronary arteries through activation of the Raf/ERK/MAPK pathway.

## Results

### ET Receptor-Mediated Contractions

The left anterior descending (LAD) coronary artery segments were examined. The contraction induced by K^+^ was used as a reference for the contractile capacity. SHS exposure *in vivo* did not affect the ability of the smooth muscle to contract in response to high K^+^-solution *in vitro* (table 1). The mean value of the K^+^ responses in fresh segments was 3.50±0.22 mN (*n* = 10) and in the smoke exposed rat LAD ring segments it was 3.80±0.34 mN (*n* = 9); there was no significant difference between the groups.

**Table 1:** Contractile response property of coronary arteries induced by K^+^, S6c and ET-1.

			S6c		ET-1	
Group	*n*	K^+^ (mN)	E_max_(% of K^+^)	pEC_50_	E_max_(% of K^+^)	pEC_50_
Fresh air	10	3.5±0.22	9.3±1.1	9.11±0.11	153±4	7.82±0.03
Smoke	9	3.8±0.34	24.4±3.1**	8.90±0.22	167±9	8.29±0.05**
Smoke+GW5074	8	4.1±0.39	18.1±3.0	9.10±0.17	162±9	7.94±0.06##

The coronary arteries were isolated from rats exposed to fresh air, cigarette smoke and cigarette smoke plus GW5074 (0.5 mg/kg). E_max_ represents maximal contraction, pEC_50_ represents the negative logarithm of the agonist concentration that produces 50% of the maximal contractile effect. Coronary artery contractile responses were induced by K^+^, S6c and ET-1. K^+^-induced responses were expressed as absolute values of contraction (mN), and S6c and ET-1-elicited responses were expressed as percentage of K^+^-induced contraction. Statistical analysis was performed with unpaired student's *t*-test with Welch's correction.

** *P*<0.01 *vs.* fresh air group;

## *P*<0.01 *vs.* smoke group.

**Figure 1:**
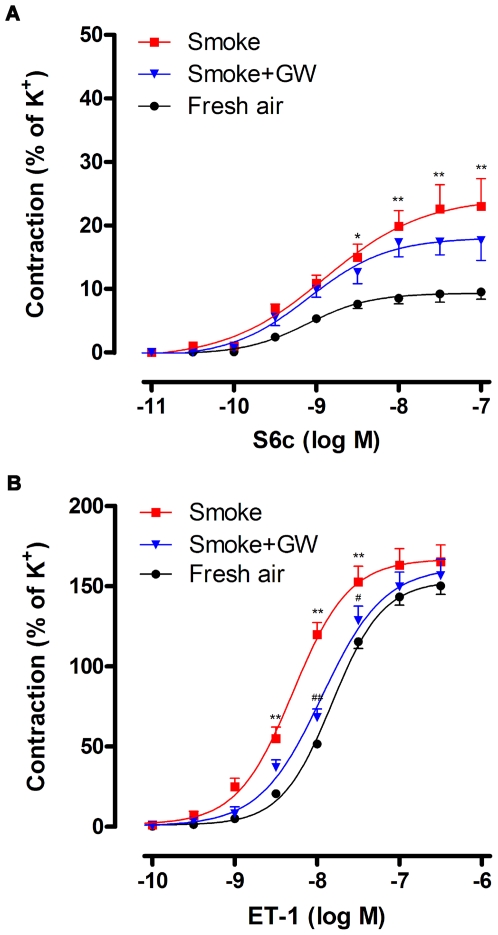
Concentration-contractile responses induced by S6c and ET-1 in rat coronary arteries. The coronary arterial segments were isolated from rats exposed to fresh air, cigarette smoke and cigarette smoke plus GW5074. Contractions were induced by cumulative application of S6c (A) or ET-1 (B). The contractile responses were shown as percentage of K^+^-induced contraction (*n* = 8–10). Statistical analysis was performed using two-way ANOVA with Bonferroni's post-test. ^*^ *P*<0.05, ^**^ *P*<0.01 *vs.* fresh air group, ^#^ *P*<0.05, ^##^ *P*<0.01 *vs.* smoke group.

In control experiments on fresh coronary arteries, the selective ET_B_ receptor agonist sarafotoxin 6c (S6c) induced a slight contraction with an E_max_ value of 9.3±1.1% (*n* = 10) relative to the K^+^ responses. SHS exposure resulted in enhanced S6c-induced contractile responses in coronary arteries (Fig. 1A). The concentration-response curve for S6c gave an E_max_ of 24.4±3.1% (*n* = 9, *P*<0.01) and a pEC_50_ of 8.90±0.22 (table 1), indicating that the efficacy of the response increased after smoke exposure. Inhibition of the Raf/ERK/MAPK pathway with GW5074 treatment did not significantly influence the contraction induced by S6c (Fig. 1A) and there was no statistical significance either in E_max_ or pEC_50_ values between the smoke and treatment groups (table 1).

ET_A_ receptor-mediated vasoconstriction was examined after desensitization of ET_B_ receptor with S6c prior to adding ET-1 (a combined ET_A_ and ET_B_ receptor agonist) [22]. Cumulative administration of ET-1 induced potent contraction in fresh coronary arteries (Fig. 1B), showing an E_max_ of 153±4% and pEC_50_ of 7.82±0.03 (*n* = 10, table 1). SHS exposure shifted the concentration-response curve toward the left with an increased pEC_50_ of 8.29±0.05 (*n* = 9, *P*<0.01, table 1). There was no significant difference of the E_max_ values between smoke and fresh air groups (table 1). After treatment with GW5074, ET-1-induced concentration-contraction curve was shifted toward the right in a parallel manner with a decreased pEC_50_ of 7.94±0.06 (*n* = 8, *P*<0.01, table 1) returning to the similar level of fresh ones, as compared to the smoke group.

### mRNA Levels of Endothelin Receptors

**Figure 2:**
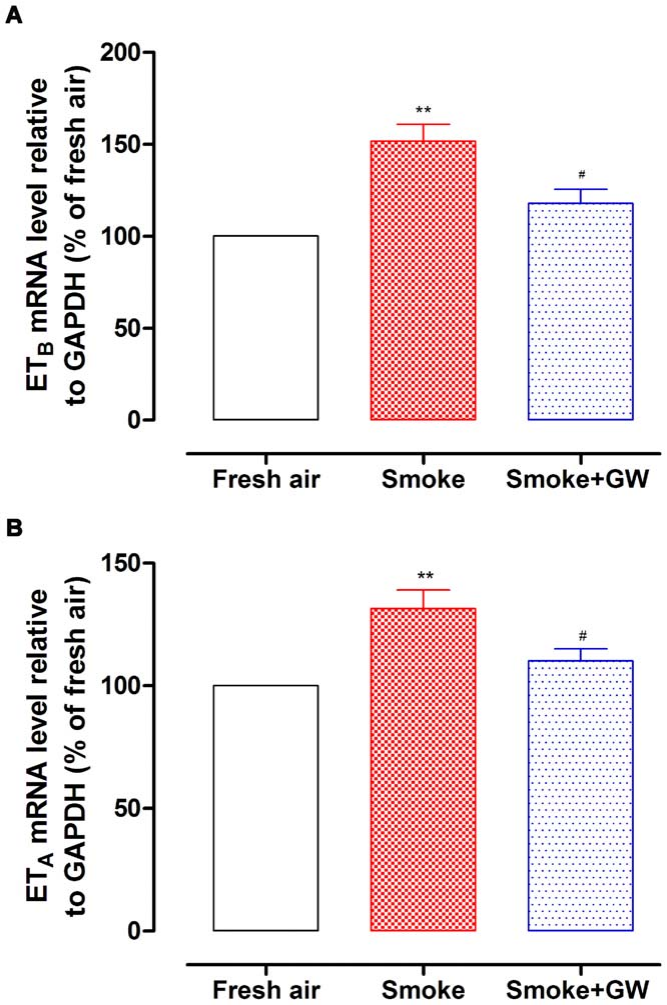
The mRNA level of ET_B_ and ET_A_ receptors in rat coronary arteries. The coronary arterial segments were isolated from rats exposed to fresh air, cigarette smoke and cigarette smoke plus GW5074. ET_B_ (A) and ET_A_ (B) receptor mRNA was relative to housekeeping gene GAPDH and shown as percentage of fresh air group by real-time PCR (*n* = 5). Statistical analysis was performed using one-way ANOVA with Dunnett's post-test. ^**^ *P*<0.01 *vs.* fresh air group, ^#^ *P*<0.05 *vs.* smoke group.

The mRNA content of ET_B_ and ET_A_ receptors in coronary arteries was examined by real-time PCR. Results showed that following smoke exposure the mRNA level of coronary artery ET_B_ receptor relative to GAPDH was increased to 152±9% (*n* = 5) of fresh air group (*P*<0.01, Fig. 2A). Similarly, ET_A_ receptor mRNA level was found significantly enhanced in coronary arteries from smoke exposed rats (132±8%, *n* = 5) as compared to the fresh air exposed ones (*P*<0.01, Fig. 2B). This is in concert with the findings on isometric contraction.

In order to demonstrate the intracellular pathway involved, we studied the inhibitory effects of Raf-1 inhibitor GW5074 on smoke exposure. Results showed that GW5074 suppressed the increased mRNA expression of both ET_B_ and ET_A_ receptors induced by smoke exposure (Fig. 2A, B). The mRNA level declined to 118±8% (ET_B_ receptor, *n* = 5, *P*<0.05) and 110±5% (ET_A_ receptor, *n* = 5, *P*<0.05), respectively, in comparison with the group exposed to smoke only.

### Protein Expression of Endothelin Receptors

**Figure 3:**
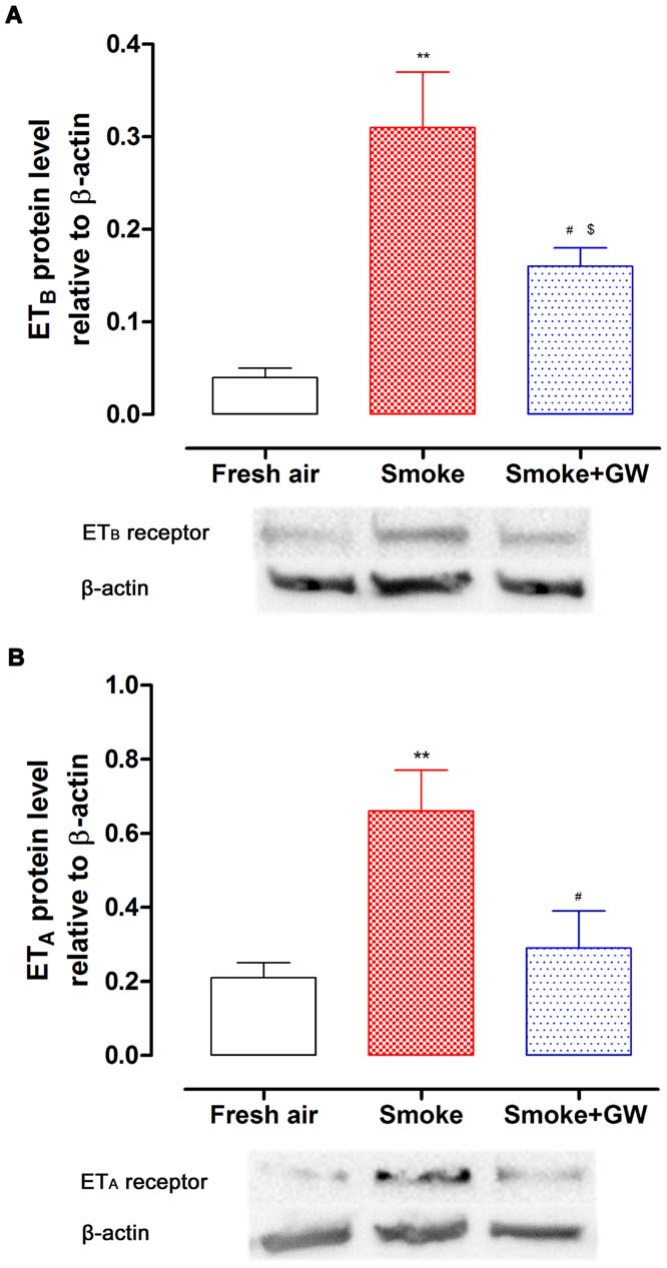
The protein expression of ET_B_ and ET_A_ receptors in rat coronary arteries. The coronary arterial segments were isolated from rats exposed to fresh air, cigarette smoke and cigarette smoke plus GW5074. ET_B_ (A) and ET_A_ (B) receptor protein was shown relative to β-actin by Western blot (*n* = 5). Statistical analysis was performed using one-way ANOVA with Dunnett's post-test. ^**^ *P*<0.01 *vs.* fresh air group, ^#^ *P*<0.05 *vs.* smoke group, *^$^P*<0.05 *vs.* fresh air group.

The protein levels of ET_B_ and ET_A_ receptors in coronary arteries were examined by Western blot. The results showed that the protein level of ET_B_ receptor relative to β-actin was 0.04±0.01 (*n* = 5) in fresh air exposed rats. In the smoke group, the ET_B_ receptor protein increased up to 0.31±0.06 (*n* = 5, *P*<0.01, Fig. 3A). The expression of ET_A_ receptor was exhibited in a similar way. Result showed that the protein level of ET_A_ receptor was 0.21±0.04 relative to β-actin in fresh air group, and it showed a markedly increase (0.66±0.11, *n* = 5, *P*<0.01) after smoke exposure (Fig. 3B). These results agree with both functional and mRNA studies shown above. Taken together, the data demonstrated that SHS exposure induces ET_B_ and ET_A_ receptor upregulation.

Western blotting was also performed in the smoke group treated with Raf-1 inhibitor GW5074. There was a significant decline in the protein level of the ET_B_ receptor (0.16±0.02, *n* = 5, *P*<0.05) in coronary arteries from rats exposed to SHS plus GW5074 treatment (Fig. 3A), compared to SHS exposure alone. However, the protein level in treatment group remained elevated from the fresh air group (*P*<0.05). For the ET_A_ receptor, the protein level was significantly decreased after inhibition of the Raf/ERK/MAPK pathway (Fig. 3B). The protein level of ET_A_ receptor relative to β-actin was 0.29±0.10 (*n* = 5) following treatment with GW5074, compared to 0.66±0.11 (*n* = 5, *P*<0.05) in the smoke group. The Raf-1 blocker GW5074 inhibited the increased receptor expression caused by smoke exposure, which supports that Raf/ERK/MAPK pathway is activated by cigarette smoke exposure.

**Figure 4:**
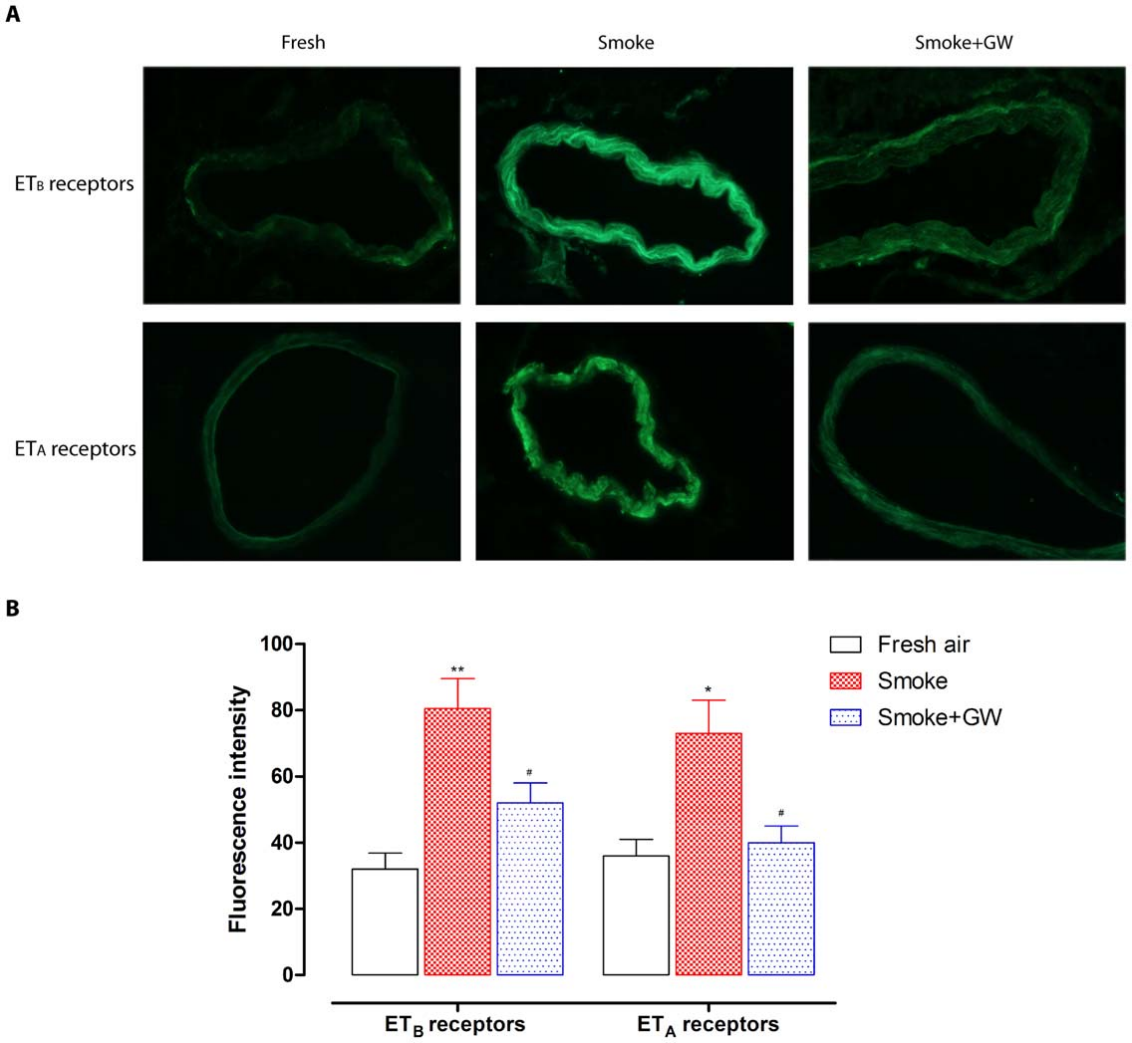
Immunofluorescence staining of ET_B_ and ET_A_ receptors in rat coronary arteries. (A) ET_B_ and ET_A_ receptor immunofluorescence in coronary arteries isolated from rat exposed to fresh air, cigarette smoke and cigarette smoke plus GW5074. (B) Bar graphs showing fluorescence intensity for ET_B_ and ET_A_ receptors. There was clear increase in ET_B_ and ET_A_ receptor protein levels in the smooth muscle cell layer of coronary arteries from smoke exposed rats, as compared to the fresh ones. Treatment with GW5074 significantly suppressed the increased receptor expression. The results were in arbitrary units of fluorescence intensity (%), a.u. Images were taken with a 40×objective (*n* = 5). Statistical analysis was performed using one-way ANOVA with Dunnett's post-test. ^*^ *P*<0.05, ^**^ *P*<0.01 *vs.* fresh air group, ^#^ *P*<0.05 *vs.* smoke group.

The ET_B_ and ET_A_ receptor protein levels were visualized by a fluorescence immunohistology method. There was a significant increase in both ET_B_ and ET_A_ receptor protein levels in the VSMC layer of SHS exposed rat coronary arteries, compared to the arteries from fresh air groups (Fig. 4A, B). Treatment with Raf-1 inhibitor GW5074 prevented the increase in expression of ET_B_ and ET_A_ receptors in the VSMCs of coronary arteries (*P*<0.05, Fig. 4A, B).

### MAPK Signal Pathway Studies

**Figure 5:**
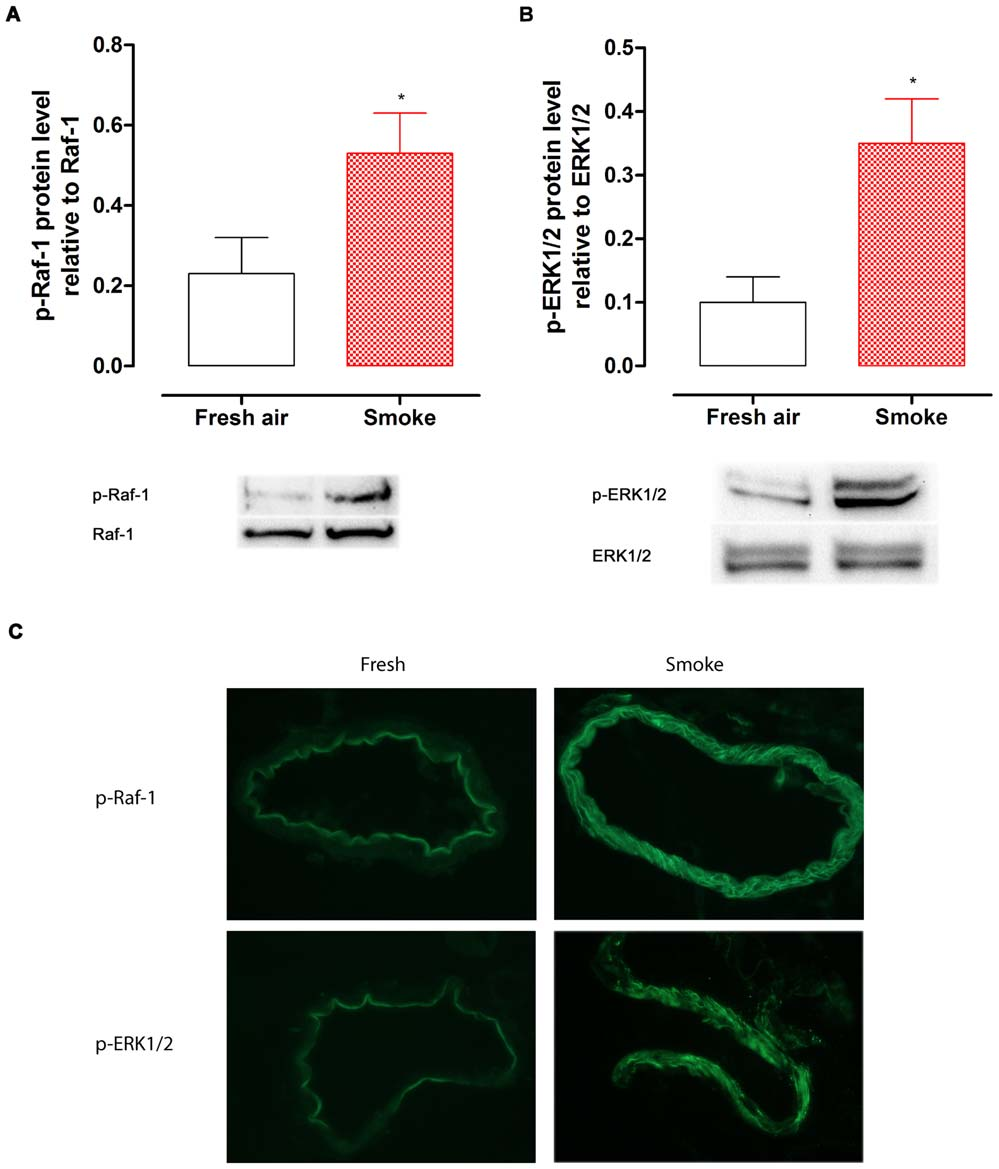
The effects of cigarette smoke on phosphorylation of Raf-1 and ERK1/2 in rat coronary arteries. The coronary arterial segments were isolated from rats exposed to fresh air and cigarette smoke. The phosphorylated (p)-Raf-1 (A) and p-ERK1/2 (B) protein was shown relative to total Raf-1 or ERK1/2 level using Western blot (*n* = 6). Statistical analysis was performed using one-way ANOVA with Dunnett's post-test. ^*^ *P*<0.05 *vs.* fresh air group. (C) Immunofluorescence staining for phosphorylation of Raf-1 and ERK1/2. There was an increase in p-Raf-1 and p-ERK1/2 expression in the smooth muscle cell layer of coronary arteries from smoke exposed rats. Images were taken with a 40×objective.

The proteins of phosphorylated (p)-Raf-1, p-ERK1/2, p-p38 and p-JNK, and their total protein expressions were examined by Western blotting in coronary arteries from rats exposed to fresh air and cigarette smoke. The results showed that the total Raf-1 and ERK1/2 proteins were at the same level in fresh air and smoke group. However, the levels of p-Raf-1 and p-ERK1/2 proteins relative to their own total protein in smoke exposed animals were 0.53±0.10 (p-Raf-1) and 0.35±0.07 (p-ERK1/2), respectively, which were both higher than that found in the fresh air group of 0.23±0.09 (p-Raf-1; *P*<0.05, *n* = 6, Fig. 5A) and 0.10±0.05 (p-ERK1/2; *P*<0.05, *n* = 6, Fig. 5B), demonstrating that cigarette smoke exposure activates Raf/ERK1/2 phosphorylation. In contrast, the phosphorylation of p38 and JNK proteins were the same in fresh air and smoke groups (data not shown). Immunofluorescence staining demonstrated that there was an increase in activated (phosphorylated) Raf-1 and ERK1/2 in the VSMC layer of coronary arteries in smoke exposed rats as compared with the ones from fresh air exposed rats (Fig. 5C). This suggests that smoke exposure-induced receptor upregulation in coronary artery occurs *via* activation of Raf/ERK1/2, but not JNK or P38 pathways.

### Relation of the receptor expression with Raf/ERK1/2 phosphorylation

Pearson's correlation analysis showed that ET_B_/ET_A_ receptor protein expression measured by Western blot positively correlated with Raf phosphorylation and ERK1/2 phosphorylation levels in rat coronary artery *in vivo*. Moreover, the receptor immunofluorescence intensity in SMC of coronary arteries was found to positively correlate with phosphorylation of Raf and ERK1/2. There were statistical significances in correlation coefficient (r) values (*P*<0.05 or *P*<0.01) (table 2). Stepwise multiple regression analysis was performed with receptor protein expression by Western blot and fluorescence intensity as dependent variables, and Raf phosphorylation and ERK1/2 phosphorylation as independent variables. The regression coefficient, β showed significant differences (*P*<0.05 or *P*<0.01) (table 2), indicating that phosphorylation of Raf and ERK1/2 were independent determinants that affected protein expression and immunofluorescence staining of ET_B_ and ET_A_ receptors.

**Table 2:** The r values of correlation analysis and β values of regression analysis between dependent variables, ET receptor protein expression by Western blot and immunofluorescence staining and independent variables, the phosphorylated (p)-Raf-1, p-ERK1/2 in rat coronary arteries.

	r values in correlation test		β values in regression test	
Dependent variables	p-Raf	p-ERK1/2	p-Raf	p-ERK1/2
ET_B_ receptor protein by Western blot	0.802**	0.774**	0.570*	0.809*
ET_B_ receptor immunofluorescence	0.854**	0.763**	1.030**	1.354*
ET_A_ receptor protein by Western blot	0.894**	0.767**	1.065**	1.344**
ET_A_ receptor immunofluorescence	0.860**	0.721*	0.871**	1.074*

Correlation analysis was performed with Pearson's correlation test and regression analysis was performed with a multiple regression test. The r value represents correlation coefficient in correlation analysis (*n* = 10) and β value represents regression coefficient in regression analysis (*n* = 10).

* *P*<0.05,

** *P*<0.01.

## Discussion

Smoking is a significant independent risk factor for CVD and is a leading cause of structural and functional alterations of the cardiovascular system [23]. It has been demonstrated that cigarette smoking is associated with vascular impairment which includes injury to the vascular endothelium, producing superoxide anions, reducing production and bioavailability of nitric oxide, increasing production and release of endothelin, causing endothelial dysfunction, atherosclerosis, which are all hallmarks of CAD [23]. The present study has for the first time demonstrated that SHS *in vivo* induces ET receptor upregulation in rat coronary arteries, and this upregulation is involved in the activation of the Raf/ERK/MAPK pathway. This may be a partial explanation of mechanisms involved in cigarette smoke-associated CAD.

Increased arterial blood pressure, heart rate and ET-1 levels were observed in smokers [24]. A previous study has already reported a significant increase in plasma ET-1 in chronic cigarette smoke exposed rats compared with non-smoking ones [11], indicating that ET-1 is associated with cigarette smoking. If an elevated ET-1 level is combined with increased number of ET receptors, then the vasoconstrictor effect in the cardiovascular system is magnified. If only the ET-1 level is increased but the receptor number not changed, there would be less possibility to see enhanced contractile responses in coronary arteries. If the receptor amount is increased, there would be a stronger contraction induced by ET-1. In other words, it is more crucial to examine the receptor number change when we study the vascular contractile property. A recent study showed that ET_A_ and ET_B_ receptor antagonists attenuated vascular hyperreactivity in aortas and carotid arteries of cigarette smoke exposed rats [11]. Tanus-Santos *et al.* also found that blockade of the ET_A_ receptor attenuated the pressor effects of nicotine inhaled in cigarette exposed rats [25], suggesting that ET receptor activation is associated with smoke-induced vascular hyperreactivity and hence involved in the pathological process of CVD. The coronary artery constriction/tone with increased resistance is more prominent in chronic smokers [26], which may contribute to the cardiovascular consequences of cigarette smoking or SHS. In the present study, we imitated the manner of SHS exposure in rats. The results showed that the smoke exposure increased contractile responses mediated by ET_A_ and ET_B_ receptors in coronary artery, but there was a need to expose the animals for 8 weeks. The choice of the time-point 8 weeks was based on a preceding study which revealed that there was no significant alteration in receptor expression at 2 or 4 weeks (data not shown). Since the contractile response reflects respective receptor expression, the receptor-mediated vasoconstriction should be in accordance with the receptor level. Interestingly, we found parallel results of mRNA and protein expressions of ET_A_ and ET_B_ receptors which are in agreement with this view. The expression of mRNA and protein for both ET_A_ and ET_B_ receptors in smoke exposed rats were higher than that in animals exposed to fresh air. The results also demonstrate that the increased ET_A_ and ET_B_ receptor expressions were primarily localized to VSMC. Accordingly, our study reveals for the first time that cigarette smoke upregulates contractile ET_A_ and ET_B_ receptors of coronary artery *in vivo*.

ET receptor activation induces strong VSMC contraction and is associated with aggravation of the progression of vessel wall hypertrophy [20]. It is known that patients with atherosclerosis have increased plasma levels of ET-1 [27]. Several studies have revealed increased number of ET_B_ receptors and stronger contractile ET_B_ receptor-mediated responses in coronary arteries in atherosclerotic lesions [8], congestive heart failure [28] and during organ culture of arteries [29]. There is a progressive increase in ET_B_ receptors in patients with suspected acute coronary syndrome and with ischemic heart disease [30], [31]. In addition, both ET_A_ and ET_B_ receptor mRNA levels have in a previous study been found to be significantly higher in coronary arteries from patients with ischemic heart disease as compared to healthy controls [10]. Collectively, this suggests that ET_A_ and/or ET_B_ receptor upregulation occurs during CAD pathogenesis. Therefore, blockage of ET receptors may offer a novel approach for the treatment and may turn out to be beneficial in vascular complications [32]. Many studies have reported that ET_A_ and ET_B_ receptor antagonists effectively reduced cardiovascular hypertrophy and decreased blood pressure in experimental hypertension [33], [34], [35]. Taken together, upregulation of ET receptors may be a key event in the development of CVD such as CAD, ischemic heart disease and atherosclerosis.

We examined the histology of coronary arteries by hematoxylin and eosin staining. The results demonstrate that there was no difference in structure between the fresh air and the smoke groups (data not shown). This suggests that our animal model of SHS exposure did not affect coronary artery structure. Nevertheless, we found contractile receptor upregulation in coronary arteries of SHS-exposed rats. The upregulated contractile receptors can lead to enhanced coronary artery vasoconstriction to ET-1, which further narrows the coronary artery lumen and reduces the blood flow. This may aggravate and facilitate the pathological process of SHS-induced coronary heart diseases. Therefore, we hypothesize that there is a close relationship between ET receptors and SHS-associated CVD.

The MAPK signal pathways are involved in a variety of cellular programs including transcription of receptors. Activation of MAPKs results in activation of transcription factors which transcribe specific receptor genes in the nucleus, which are then translated in the cytoplasm and exported to the cell membrane [36]. In previous studies we have shown that the MEK/ERK pathway is involved in the receptor upregulation, in particular related to stroke [37]. Recently, we have revealed that cigarette smoke extracts *in vitro* promote VSMC proliferation and enhance contractile responses in vasculature *via* activation of ERK1/2 signaling [6]. In the present study, SHS exposure increased the proportion of p-Raf-1 and p-ERK1/2 protein in coronary arteries, indicating that the Raf/ERK/MAPK pathway may be involved in the process of the ET receptor upregulation, but not the p38 or the JNK pathways. Furthermore, our data of correlation analysis in the present study showed significant positive correlations between receptor (ET_B_ and ET_A_) protein expression levels and Raf/ERK1/2 phosphorylation. On the other hand, regression analysis showed that phosphorylation of Raf and ERK1/2 were independent factors to impact protein expression level and immunofluorescence intensity of ET_B_ and ET_A_ receptors. These results suggest that the activation of the Raf/ERK/MAPK pathway is closely linked to the receptor expression in coronary arteries *in vivo*.

Raf-1 is a MAP kinase kinase kinase, which functions downstream of the Ras family of protein kinases [18]. Activated Raf-1 can phosphorylate MEK1 and MEK2, which in turn activate ERK1 and ERK2. Downstream of Raf-1, activated ERK1/2 plays an important role in the control of gene expression. In the present study a Raf-1 inhibitor GW5074 was chosen to further prove the involvement of Raf/ERK/MAPK pathway in the upregulation. The specificity and efficacy of GW5074 for inhibiting Raf-1 *in vivo* has been established in previous studies [38]. The present results showed that GW5074 attenuated the SHS-induced elevated coronary contraction mediated by ET_A_ receptors and the receptor mRNA and protein levels. In addition, GW5074 decreased the smoke exposure-induced increase in mRNA and protein levels of ET_B_ receptors. This strongly suggests that the SHS-induced upregulation of ET_A_ and ET_B_ receptors in coronary artery is associated with the activation of the Raf/ERK/MAPK signaling.

To conclude, cigarette smoke exposure upregulates ET_A_ and ET_B_ receptors of rat coronary arteries, which is related to the activation of the Raf/MEK/ERK pathway. This may provide a new prospective on possible mechanisms involved in SHS-associated CAD.

## Materials and Methods

### Animals

Experiments were performed on male Sprague-Dawley rats (200–250 g) supplied by the Animal Center of Xi'an Jiaotong University College of Medicine (Xi'an, China). Animals were allowed free access to fresh food and water. All experimental protocols were endorsed by the Animal Ethics Committee of Xi'an Jiaotong University.

### Cigarette Smoke Exposure Model

Thirty rats were randomly divided into 3 groups, 10 rats in each group: (1) fresh air exposure; (2) smoke exposure; (3) smoke exposure and treatment with GW5074 0.5 mg/kg. GW5074 was a gift from professor Yu-hai Tang (Science College of Xi'an Jiaotong University, China) and its specificity has previously been studied [38]. The rats were exposed to cigarette smoke from commercially-available filter cigarettes (Marlboro, 1.0 mg of nicotine and 12 mg of tar content) in a plastic chamber (115×50×65 cm; 0.37 m^3^). Ten rats were put in the chamber and each cigarette was lit from the cigarette end and freely burning for 15 min, then let the cigarette smoke diffuse in the whole chamber for another 25 min. Fresh air inhalation was performed for at least 10 min after every 40 min of SHS exposure [39]. For each smoke exposure, 2 cigarettes were lit simultaneously. The rats were repeatedly exposed to the smoke for total 10 cigarettes every day for up to 8 weeks. Total time of smoke exposure was 5×40 min = 200 min/day. Gas chromatography was used to determine the CO_2_ concentration inside the chamber. It was 774±85 ppm at the time point of burning for 15 min and 611±89 ppm when the smoke diffusing for another 25 min, respectively. The rest of time animals were exposed to fresh air. In fresh air group, rats were placed in similar chambers and exposed to room air. For the treatment group, GW5074 was administrated intraperitoneally to the animals once every day for 8 weeks in addition to the same condition of smoke exposure.

### Coronary Artery Harvest

The rats were anesthetized with CO_2_ and decapitated to prepare artery samples 24 h after the final exposure. The heart was immediately removed and immersed into cold bicarbonate buffer solution [40] from each rat. The LAD coronary arteries, left circumflex arteries and right coronary arteries were isolated from the heart. Some LAD arteries were cut into segments for myograph function examination. Some LAD arteries were fixed in 4% paraformaldehyde overnight for immunofluorescence analysis. The other LAD arteries as well as circumflex arteries and right coronary arteries were snap frozen at −80°C for real-time PCR and Western blot studies.

### Coronary Artery Contractile Studies

The *in vitro* contractility experiments were performed in the Xi'an Jiaotong University, while the other experiments were done in the Lund University. In the present study, wire myograph (Danish Myo Technology A/S, Aarhus, Denmark) was used for recording the vessels contractile properties. The LAD arteries were cut into ring segments of approximately 1–2 mm in length and then mounted on two thin wires (40 µm in diameter). The set-up has been detailed described before [29]. The rings were allowed to equilibrate for at least 1.5 h with an optimal resting tension of 1.0 mN. Thereafter, the viability of the arterial segments was determined by exposure to 63.5 mM K^+^ buffer solution.

Concentration-contraction responses were obtained by cumulative administration of contractile receptor agonists S6c (Merck, Darmstadt, Germany), and ET-1 (NeoMPS, Strasbourg, France). The ET_B_ receptor-mediated contractile response was obtained by using the ET_B_ receptor selective agonist S6c. When the contraction reached the maximum, i.e. desensitization of ET_B_ receptor, ET-1 (both ET_A_ and ET_B_ receptor agonist) was then administrated to induce a contraction response that was only mediated by ET_A_ receptors [40].

### Real-time PCR

Total RNA was extracted from the collected coronary arteries using RNeasy Mini kit (Qiagen, Valencia, CA) with the instruction of the supplier. The RNA concentration was measured and recorded with an Eppendorf Biophotometer (Hamburg, Germany). Reverse transcription of total RNA to cDNA was performed using the TaqMan Reverse Transcription Reagents (Applied Biosystems, Branchburg, USA) in a Perkin Elmer 2400 GeneAmp PCR system (Perkin Elmer, MA, USA). Real-time PCR was carried out using the GeneAmp SYBR® Green kit (Applied Biosystems) in a GeneAmp 7300 Sequence Detection System (Applied Biosystems). The system automatically monitors the binding of a fluorescent dye SYBR® Green to double-stranded DNA during each cycle of PCR amplification. The real-time PCR was prepared in 25 µl reaction volume and started at a temperature of 50°C for 2 min; 95°C for 10 min and the following 40 thermal cycles with 95°C for 15s and 60°C for 1 min. The dissociation curves were ran after the real-time PCR to identify the specific PCR products. A blank without template was included in all the experiments as negative controls. Specific primers for the receptors were designed as follows: ET_A_ receptor, forward: 5′-GTC GAG AGG TGG CAA AGA CC-3′, ET_A_ receptor, reverse: 5′-ACA GGG CGA AGA TGA CAA CC-3′; ET_B_ receptor, forward: 5′-GAT ACG ACA ACT TCC GCT CCA-3′, ET_B_ receptor, reverse: 5′-GTC CAC GAT GAG GAC AAT GAG-3′. As an endogenous standard, the primers of glyceraldehyde-3-phosphate dehydrogenase (GAPDH) were designed as follows: GAPDH, forward: 5′-GTC CAC GAT GAG GAC AAT GAG-3′, GAPDH, reverse: 5′-CGG CAT GTC AGA TCC ACA AC-3′. The comparative cycle threshold (C_T_) method was used for the mRNA content analysis [41], [42].

### Western Blot

The coronary arteries were harvested as mentioned above, snap frozen in liquid nitrogen. The arteries were homogenized in cell extract denaturing buffer (BioSource, USA) with addition of a phosphatase inhibitor cocktail and protease inhibitor cocktail (Sigma, St. Louis, USA). The mixtures were then centrifuged at 12,000×g at 4°C for 10 min and the supernatants were collected as protein samples. Protein concentrations were measured by using a protein assay reagent (Bio-Rad Laboratories, Hercules, CA) and a Tecan Infinite M200 microplate reader. For western blotting, protein samples were mixed with Laemmli Sample Buffer (Bio-Rad Laboratories) and boiled for 5 min. Equal amount of protein (40 µg) were loaded onto a 4–15% Ready Gel Precast Gels (Bio-Rad Laboratories). A molecular weight marker was loaded onto each gel for protein band identification. After gel eletrophoresis the proteins were transferred to a nitrocellulose membrane (Bio-Rad Laboratories). The membrane was then blocked in 5% non-fat milk and incubated with the primary antibodies at appropriate dilution at 4°C overnight. The antibodies used were shown as follows: 1∶200 rabbit anti-ET_A_ receptor (sc-33535, Santa Cruz Biotechology, CA, USA), 1∶500 rabbit anti-ET_B_ receptor (ab65972, Abcam, Cambridge, UK), 1∶1000 rabbit anti-phospho-c-Raf (Ser338) (#9427, Cell Signaling Technology, Beverly, CA), 1∶2000 rabbit anti-phospho-ERK1/2 (#4370, Cell Signaling Technology), 1∶1000 rabbit anti-phospho-SAPK/JNK (#4668, Cell Signaling Technology), 1∶1000 rabbit anti-phospho-p38 (#4631, Cell Signaling Technology), 1∶1000 mouse anti-β-actin (#4970, Cell Signaling Technology) or 1∶2000 mouse anti-ERK1/2 (#4696, Cell Signaling Technology). Then, membranes were incubated with 1∶2000 horseradish peroxidase-conjugated anti-rabbit (#7074, Cell Signaling Technology) or anti-mouse (#7076, Cell Signaling Technology) secondary antibodies for 1 h at room temperature. Finally, membranes were developed and visualized using a Fujifilm LAS-1000 Luminescent Image Analyzer (Fujifilm, Stamford, USA) and the band intensity was quantified using ImageJ software.

### Immunofluorescence

The paraformaldehyde-fixed arteries were embedded in paraffin and cut into 4-µm sections. After deparaffinization, the sections were treated with 10 mM sodium citrate buffer, pH 6.0, in a microwave oven for 10 min for antigen retrieval. Immunostaining with rabbit polyclonal antibody ET_A_ receptor (sc-33535, Santa Cruz Biotechology) and ET_B_ receptor (sc-33538, Santa Cruz Biotechology) diluted 1∶100, rabbit monoclonal phosphor-Raf-1 (Ser338) antibody (#9427, Cell Signaling Technology) and phospho-ERK1/2 antibody (#4370, Cell Signaling Technology) diluted 1∶100 were performed. Goat anti-rabbit IgG conjugated to fluorescence isothiocyanate (FITC, #10006588, Cayman Chemical, Michigan, USA) diluted 1∶100 was used as secondary antibody. Then the slides were sealed with anti-fading mounting medium. To identify the smooth muscle layer of the artery segments, immunostaining with the primary antibody against rat smooth muscle actin (sc-53015, Santa Cruz Biotechology) diluted in 1∶200, and the secondary antibody donkey anti-mouse IgG (H+L) conjugated to Texas Red (#715-076-150, Jackson ImmunoResearch) diluted in 1∶200 were also performed. In the control experiments, either the primary antibody or the secondary antibody was omitted. Immunoreactivity was visualized with an epifluorescence microscope (Nikon 80i, Tokyo, Japan) and photographed using a digital camera (Nikon DS-2Mv).

### Calculations and Statistics

All data are expressed as means ± S.E.M. and *n* refers to the number of rats. Contractile responses to agonists in each segment were expressed as percentage of the K^+^-induced contraction. E_max_ values represent maximal contractile response elicited by agonists and pEC_50_ values represent the negative logarithm of the agonist concentration that produces 50% of the maximal contractile effect. Unpaired Student's *t*-test was applied to compare two sets of data. One-way analysis of variance (ANOVA) with Dunnett's post-test was used for multiple comparisons. Two-way ANOVA with Bonferroni's post-test was performed to compare the two corresponding data points at each concentration of the two curves. Pearson's correlation analysis was used to determine the relationship between receptor protein expression and Raf/ERK1/2 phosphorylation. Stepwise multiple regression analysis was performed to define the factors of influencing the receptor protein expression and fluorescence intensity in SMC of coronary arteries. All calculations and statistical analysis were carried out using Graph-Pad Prism 5.0 (GraphPad Software, La Jolla, USA). Statistical significance was accepted when *P*<0.05.
